# Dynamic response of pre-disintegrated carbonaceous mudstone embankment under multi-lane vehicle load

**DOI:** 10.1371/journal.pone.0270937

**Published:** 2022-07-07

**Authors:** Zhongming He, Panpan Wang, Weidi Gong

**Affiliations:** 1 School of Traffic and Transportation Engineering, Changsha University of Science and Technology, Changsha, Hunan, China; 2 Guangxi Xinfazhan Communication Group Co., Ltd, Nanning, Guangxi, China; Tongji University, CHINA

## Abstract

The purpose of this study is to reveal the response of multi Lane pre disintegrated carbonaceous mudstone embankment under vehicle dynamic load. In this paper, the pre-disintegrated carbonaceous mudstone samples whose fractal dimension meets the requirements are obtained through the indoor disintegration test of carbonaceous mudstone. Geotechnical basic tests such as particle analysis experiments, compaction tests, and direct shear tests were carried out on the pre-disintegrated carbonaceous mudstone samples, and the physical and mechanical parameters of the pre-disintegrated carbonaceous mudstone were obtained. On this basis, a two-way 4-lane pre-disintegration carbonaceous mudstone embankment model of the expressway was established by ABAQUS numerical software. Three different working conditions are set up to study the dynamic response of multi-lane pre-disintegrated carbonaceous mudstone embankment under vehicle load. The results show that the stress change trend on the surface of the pre-disintegrated carbonaceous mudstone embankment without vehicles is the same as that on the side with vehicles. Under this condition, the vertical displacement of the pre-disintegrated carbonaceous mudstone embankment surface can be as high as 4.33mm, and the vertical displacement change of the embankment in the 0–0.6s phase is basically the same as the stress amplitude distribution. When a traffic jam occurs on one side, the maximum increase in vertical stress on the surface of the embankment on the normal driving side is about 170 kPa compared to condition one, and the vertical displacement at each depth of the embankment has been significantly increased. When a traffic jam occurs on one side, it can significantly increase the vertical stress on the surface of the pre-disintegrated carbonaceous mudstone embankment in this lane. The middle part of the stress time curve of monitoring points 3 and 4 in working condition three is more stable and significant than in working condition one, and the maximum vertical displacement is increased by about 1.70mm. The research results can reference the stability analysis of carbonaceous mudstone embankments and engineering practice.

## 1. Introduction

Carbonaceous mudstone is widely distributed in rainy areas in western and southern China [1, 2], especially in Guangxi. Carbonaceous mudstone is a geological body composed of sedimentary rocks such as soft limestone, sandstone, and shale. Carbonaceous mudstone is black because the sedimentary rock is rich in carbon, and has the characteristics of easy disintegration, reduced strength, and increased deformation when exposed to water [3]. Due to the wide distribution of carbonaceous mudstone, it has become an important topic in the research of carbonaceous mudstone to make reasonable use of carbonaceous mudstone as road embankment fill to maximize its economic benefits.

At present, many scholars have carried out much research on carbonaceous mudstone. Fu Hongyuan and Zeng Ling [4–7] revealed the strength change principle of pre-disintegrated carbonaceous mudstone under three triaxial computed tomography (CT) synchronous scanning and the indoor conventional geotechnical tests and obtained the influence law of cycle times and vertical loading on the shear strength of pre-disintegrated carbonaceous mudstone. Liu Ziang [6] conducted direct shear tests on mixed soils with different contents of carbonaceous mudstone and concluded that the higher the content of carbonaceous mudstone, the smaller the internal friction angle of the mixed soil and rock, and the greater the cohesion. Ye Chaoliang [8] conducted indoor dynamic model tests to obtain the dynamic deformation law of the carbonaceous mudstone at the pile tip before and after water immersion and under dynamic load. Liu Xinxi [9] used FLAC3D software to calculate the slope safety factor. It is suggested to calculate the slope safety factor by combining the rheological characteristics of soft carbonaceous mudstone interlayer and to use the long-term stability coefficient of slope as the design parameter before support.

For the research on the dynamic response of the embankment, scholars have long used field tests and model calculations. With the advent of informatization and the advancement of science and technology, people began to use finite element software related to road engineering to analyze the dynamic response of embankments and related issues, and the results were abundant. Based on the elastic plasticity theory and creep theory, Wang tiehang [10] established a two-dimensional model to calculate the stress and deformation of highway embankment in the permafrost region by simulating the variation of water content, temperature distribution, and soil properties. Zhang Mingyi [11] analyzed the critical embankment height (coarse-grained soil) in the perennial permafrost zone of the Qinghai-Tibet Plateau using the finite element method. Shaharaki [12] investigated the dynamic response of the track and embankment in the transition section between unballasted and ballasted track using ANSYS software. Fernández [13] established a three-dimensional numerical model of the embankment and studied the environmental vibration caused by the train through PLAXIS software. By comparing the actual measurement data with the model calculation results, the correctness of the established model is verified, and the application scope of PLAXIS software was further expanded. Yang [14, 15] introduced the 2.5D numerical simulation technology into calculating the dynamic response of three-dimensional foundation under trainload and derived the 2.5D finite element governing equations of viscoelastic foundation and elastic foundation under moving trainload. Costa [16] considered the influence of the nonlinear characteristics of the foundation material on the dynamic response of the embankment in 2.5D finite element and analyzed the distribution of the dynamic modulus reduction coefficient of the foundation under the action of different vehicle speeds, and pointed out that the greater the vehicle speed, the greater the influence of nonlinear characteristics of foundation material on calculation results. Pang Feng [17] used ABAQUS finite element analysis method to analyze the dynamic characteristics of low highway embankments under vehicle load. Li Youyun [18] established a three-dimensional finite element calculation model to analyze and calculate the force of the loess embankment structure under the vehicle load under three working conditions. Qiu Chengchun [19] and others established a two-dimensional numerical model of the embankment using particle flow software based on discrete element theory to analyze the dynamic response of different reinforced embankments under vehicle load. Dong Liancheng [20] used ABAQUS to establish a typical cross-section model of the Ha-Jia railway and studied the variation law of seismic acceleration and final displacement along with the height of the filled embankment. Zha Wenhua [21] took Jiangsu Lianyan Expressway as the research object, compared the actual measured value with the numerical simulation solution, and verified the feasibility of the coupled dynamic model embankment and the specific calculation method he established. In summary, the dynamic response of vehicle load to embankment can be accurately analyzed by using numerical simulation software [22, 23]. Moreover, although many scholars have done a lot of research on carbonaceous mudstone, they mainly focus on the geological characteristics of carbonaceous mudstone, and there is still a lack of research on the dynamic response of carbonaceous mudstone embankment. Therefore, it is necessary to use numerical simulation software to study the dynamic response of carbonaceous mudstone embankment, so as to provide a reference for the stability analysis of carbonaceous mudstone embankment.

ABAQUS is one of the most powerful finite element software in the world. It can simulate very complex conditions and deal with highly nonlinear problems. Its computational reliability has been widely recognized. Therefore, ABAQUS software is selected to study the dynamic response of carbonaceous mudstone embankment under a multi-lane traffic load. In addition, since carbonaceous mudstone is a soft rock rich in organic matter, its most important characteristic is that it will disintegrate when it meets water. In practical engineering, if the disintegration fraction dimension of carbonaceous mudstone reaches 2.3~2.5, it is considered that the carbonaceous mudstone has been stably disintegrated and can be used for embankment filling at this time. Taking into account this, this article takes the pre-disintegrated carbonaceous mudstone embankment as the research object. Firstly, the physical and mechanical properties of the pre-disintegrated carbonaceous mudstone were studied through soil tests. Then, depending on the physical and mechanical indexes of the pre-disintegrated carbonaceous mudstone, we used the numerical simulation software ABAQUS to study the dynamic response characteristics of the multi-lane pre-disintegrated carbonaceous mudstone embankment under vehicle load. The dynamic response characteristics of the multi-lane pre-disintegrated carbonaceous mudstone embankment under three different working conditions are compared and analyzed, which provides theoretical guidance for the popularization and application of carbonaceous mudstone in highway engineering.

## 2. Establishment of pre-disintegrated carbonaceous mudstone embankment model

### 2.1 Determination of physical and mechanical parameters of pre-disintegrated carbonaceous mudstone

This article first conducts an indoor disintegration test of carbonaceous mudstone to make its fractal dimension between 2.3 and 2.6. Then, particle analysis experiments, compaction tests, and direct shear tests were carried out on the carbonaceous mudstone samples satisfying the fractal dimension [24, 25]. Through the above tests, the physical and mechanical parameters of the pre-disintegrated carbonaceous mudstone were obtained, as shown in Table 1, in preparation for the later numerical simulations.

**Table 1:** Physical and mechanical parameters of pre-disintegrated carbonaceous mudstone.

Non-uniformity coefficient (*C*_u_)	Curvature coefficient (*C*_c_)	Maximum dry density (*ρ*_*d*_) / (*g*/*cm*^3^)	Optimal moisture content (*ω*_*op*_) /%	Cohesion c /kPa	Internal friction angle (°)
6.09	1.51	2.12	7.50	28	13.6

### 2.2 Description of working conditions and establishment of finite element model

#### 2.2.1 Description of working conditions

With reference to relevant research materials and literature, most scholars only consider the single-lane when studying the dynamic response of vehicle loads to the embankment and only consider half of the embankment in a symmetrical state when setting up the model. However, these conditions are not in line with reality. Therefore, a numerical model of a two-way four-lane pre-crumbling carbonaceous mudstone road embankment in the north-south direction is developed in this paper to study the dynamic response of the pre-crumbling carbonaceous mudstone embankment under the traffic load.

In this paper, a single axle two-wheel set vehicle with 100kN axle load is selected, with a single wheel load p = (100/4) kN and tire pressure 700kPa. In this paper, in order to simulate the normal driving state and congestion state of vehicles, different numbers of vehicles and vehicle speed are set up on the two-way lanes, and three working conditions with three different vehicle traffic states were established. The normal driving speed of the vehicle is 100 km/h, and the driving speed of the vehicle in congestion is 5 km/h. The three working conditions are shown in Table 2.

**Table 2:** Description of three working conditions.

Lane	Working condition one	Working condition two	Working condition three
Northbound lane	4 vehicles (100km/h)	4 vehicles (100km/h)	4 vehicles (100km/h)
Southbound lane	0vehicle	12 vehicles (5km/h)	4 vehicles (100km/h)

### 2.2.2 Establishment of finite element model

**Fig 1:**
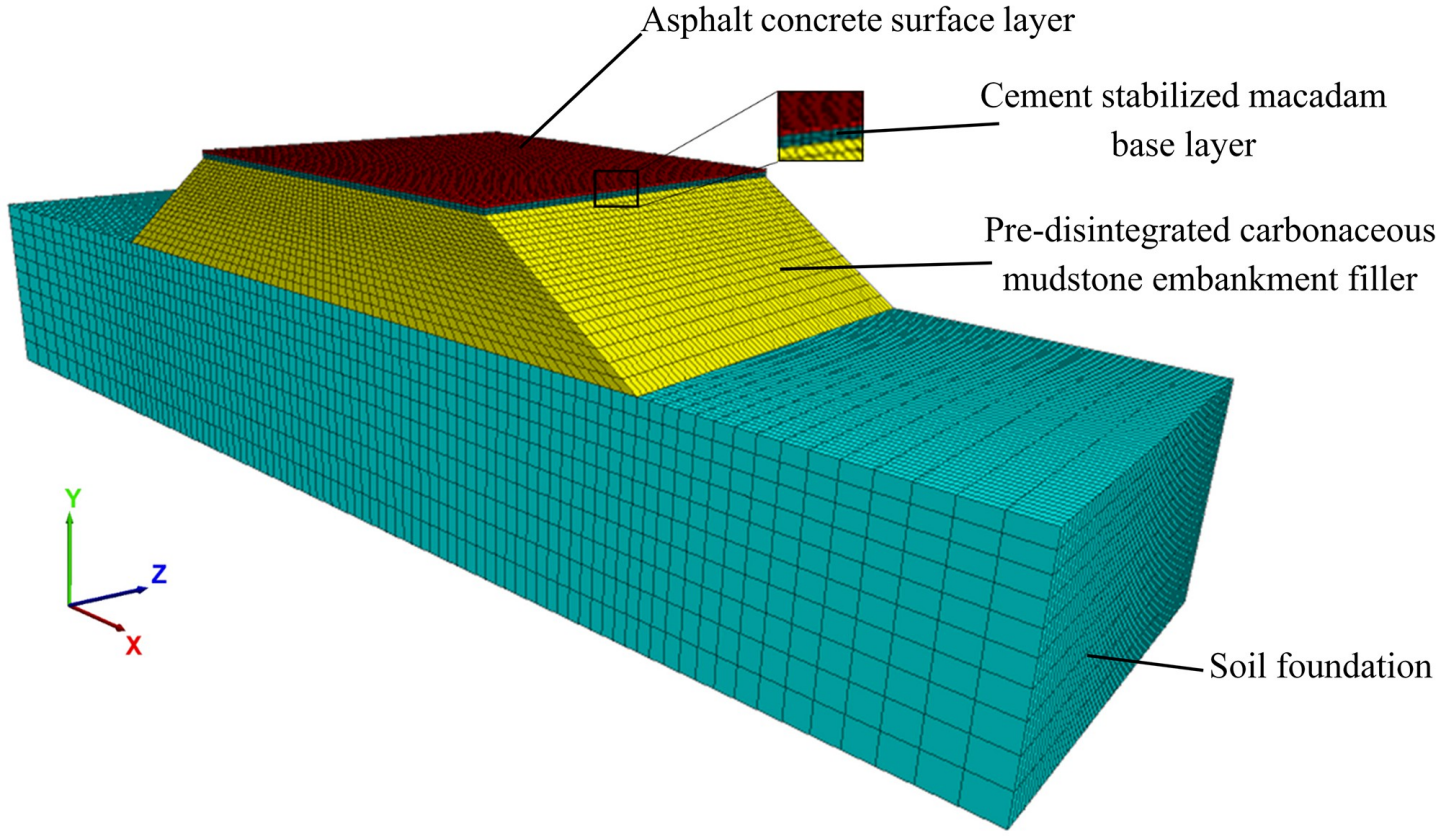
Pavement structure model diagram.

**Fig 2:**
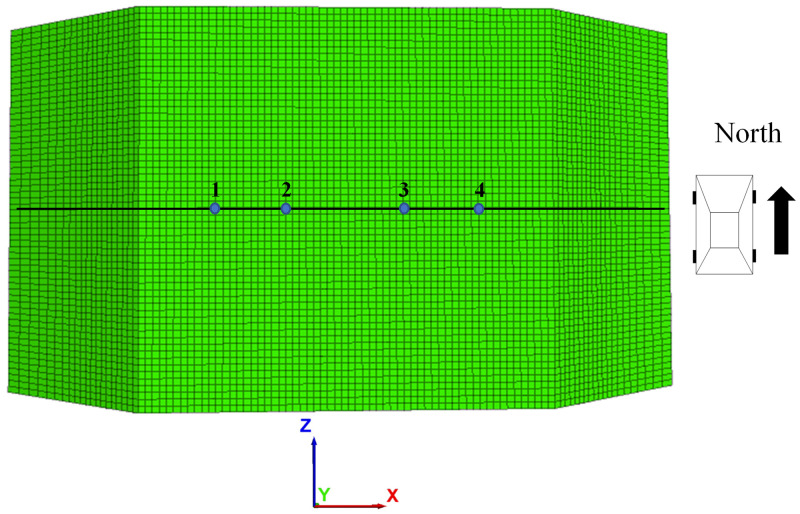
Schematic layout of measurement points.

In this paper, a two-way four-lane pre-disintegrated carbonaceous mudstone embankment model is established [10, 26]. The X-direction of the model is the cross-section direction of the road with a length of 100m, the Y direction is the height direction of the embankment with a height of 18.58m, and the Z direction is the direction of the longitudinal road section with a length of 30m. The pavement structure used in the numerical simulation is a common highway pavement structure in southern China, as shown in Fig 1. The embankment model is divided into four layers: 18cm thick asphalt concrete surface layer, 40cm thick cement stabilized macadam base layer, 8m thick pre-disintegrated carbonaceous mudstone embankment filler, and 10m thick soil foundation. The side slope of the embankment is a one-story side slope, in which the slope height is 8m, and the slope ratio is 1:1.5. The calculation parameters of each layer of embankment and pavement structure are shown in Table 3. The contact area between tire and road surface is 0.036m^2^. The boundary conditions of this model are vertical and horizontal constraints at the bottom, the free boundary at the top, horizontal constraints around, and the free surface at the embankment slope. Take the center position of each lane on the roadbed surface as the observation point, and take a total of four observation points. Fig 2 shows a simple schematic of the location of the measurement points.

**Table 3:** Calculation parameters of embankment and pavement structure.

Material parameter	Thickness (cm)	Cohesion (kPa)	Internal friction angle (°)	Bulk density (kN/m^3^)	Elastic modulus (MPa)	Poisson’s ratio	Damping ratio
Surface layer	18	—	—	23	1200	0.25	0.05
Base layer	40	—	—	26	2500	0.25	0.05
Embankment	800	28	13.6	21	270	0.28	0.05
Soil foundation	1000	15	20	19	30	0.4	0.05

## 3. Analysis of the influence of vehicle load on embankment stress under three working conditions

### 3.1 Analysis of stress change of embankment and monitoring points under the action of vehicle load in working condition 1

**Fig 3:**
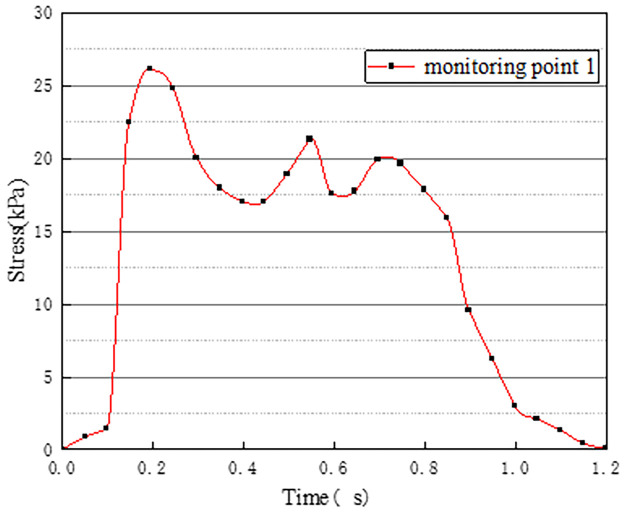
Stress time history curve of monitoring point 1.

**Fig 4:**
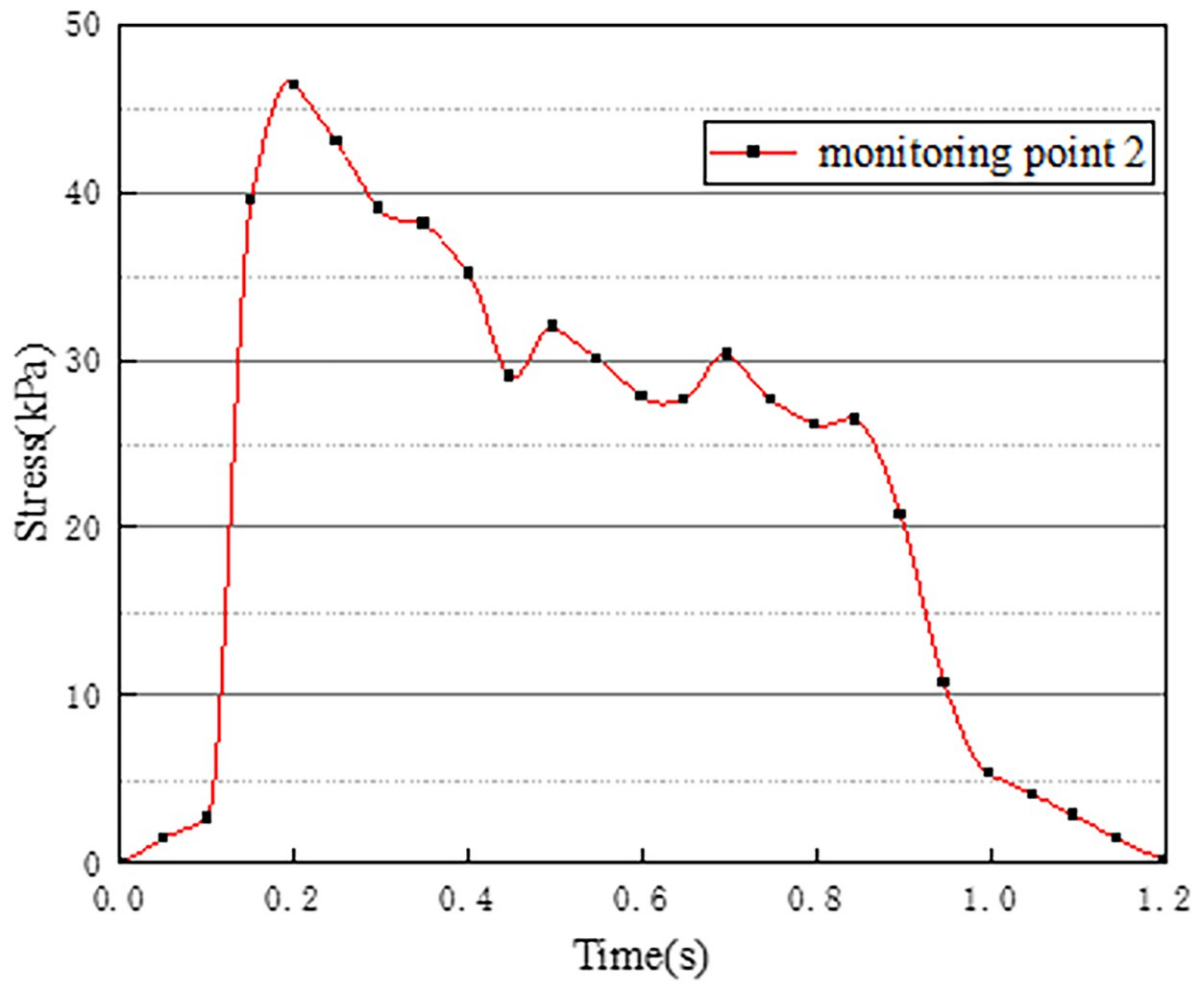
Stress time history curve of monitoring point 2.

**Fig 5:**
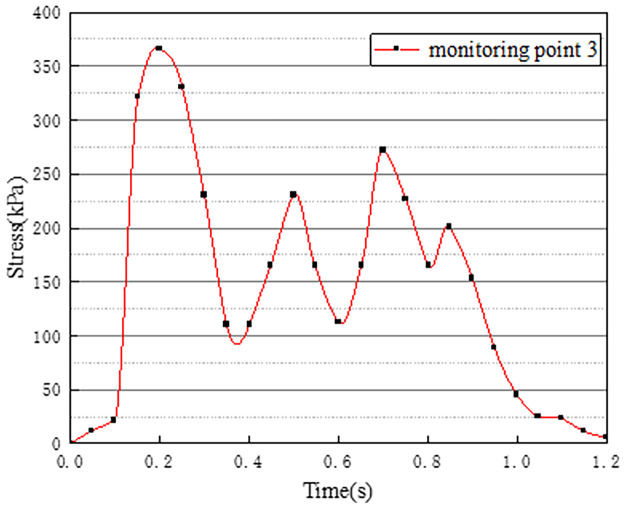
Stress time history curve of monitoring point 3.

**Fig 6:**
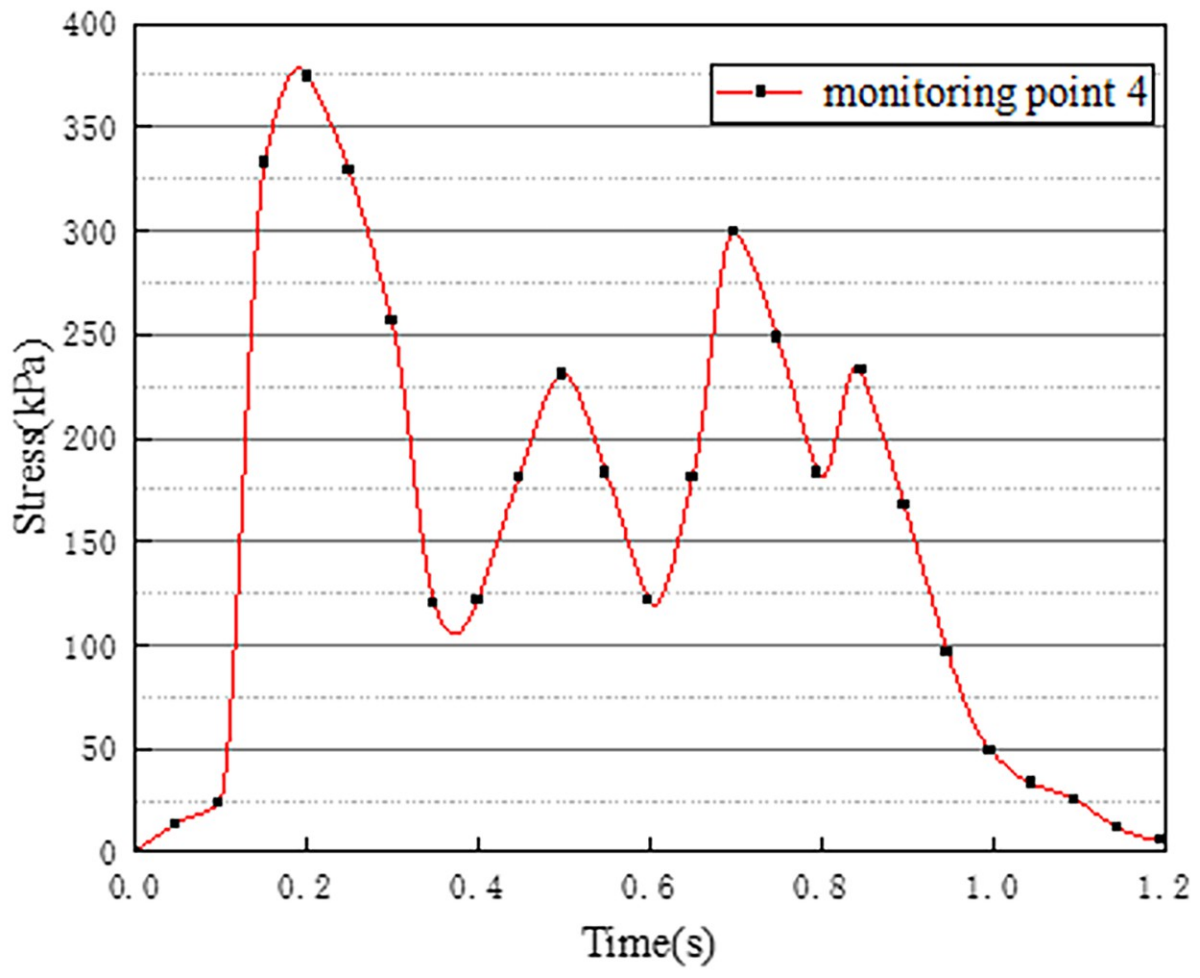
Stress time history curve of monitoring point 4.

In working condition one, the northbound lane is set to drive normally with cars, and the southbound lane is without cars. The effect of vehicle load on the vertical stress on the surface of the pre-disintegrated carbonaceous mudstone roadbed is studied. The stress curves of four different monitoring points with time are shown in Figs 3–6.

Figs 3–6 show the stress time history curves of four measuring points on the top surface of the roadbed under working condition 1. Monitoring points 1 and 2 are located in the southbound lanes without cars. The maximum stress on monitoring point 1 is 26kPa, and the maximum stress on monitoring point 2 is 46kPa. It can be obtained that the southbound lane is also affected by the vehicle load of the adjacent lane when there is no vehicle, and the closer the measurement point is to the vehicle load of the adjacent lane, the greater the stress. It can be seen from the stress time history curves of measuring points 3 and 4 that when there are two vehicle loads, there are four stress peaks, and the maximum peak value is 373kPa. The time interval between the last two peaks is shorter than the time interval between the first two peaks, which may be caused by the hysteresis of stress transmission in the soil layer. Comparing and analyzing the time history curves at points 1, 2, 3, and 4, we can get that the stress change law of the lane without vehicles is basically the same as that of the lane with vehicles.

### 3.2 Analysis of stress changes in embankment and monitoring points under vehicle load in working condition 2

**Fig 7:**
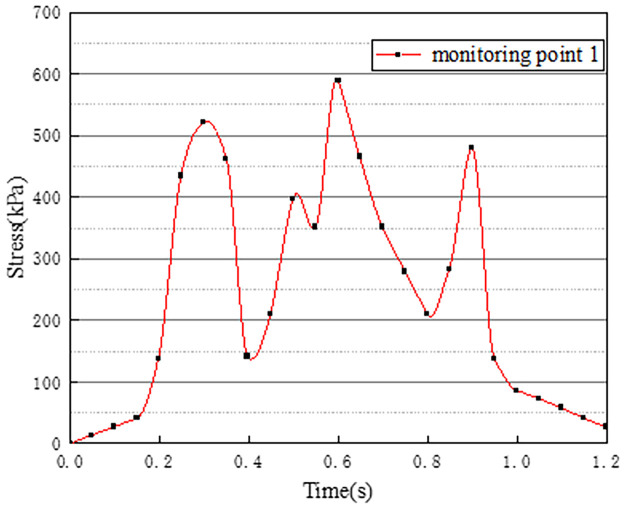
Stress time history curve of monitoring point 1.

**Fig 8:**
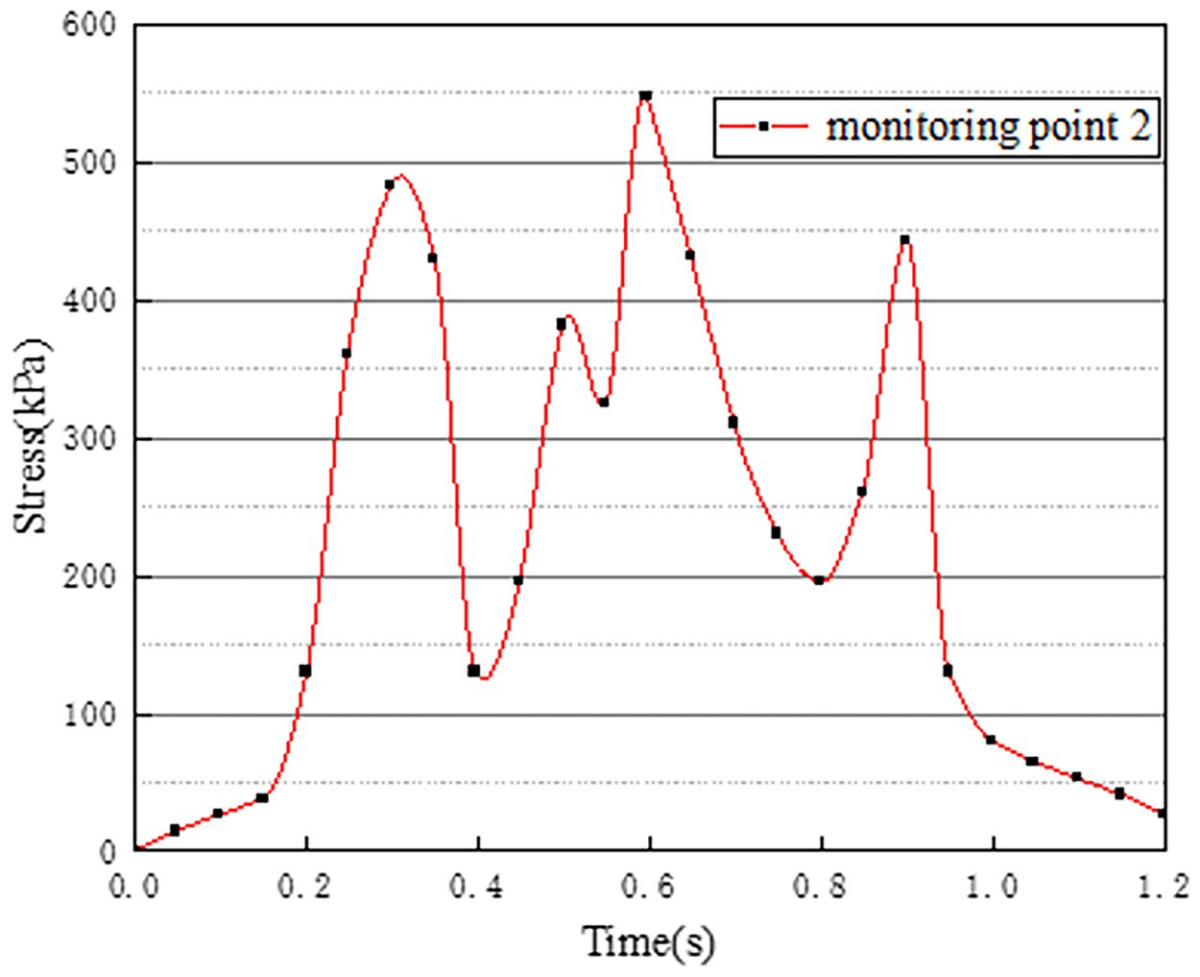
Stress time history curve of monitoring point 2.

**Fig 9:**
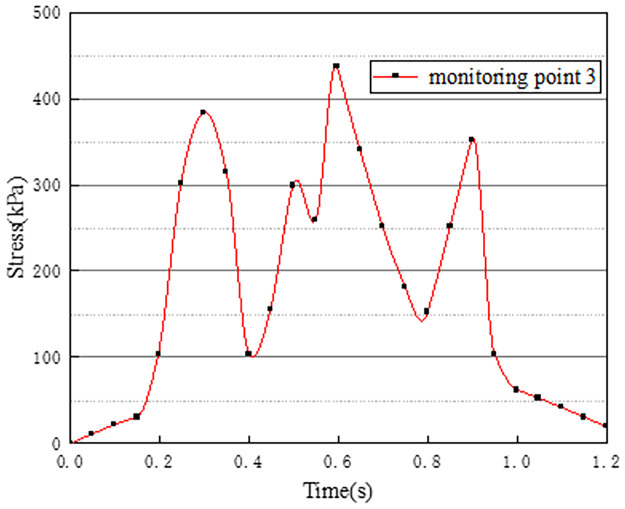
Stress time history curve of monitoring point 3.

**Fig 10:**
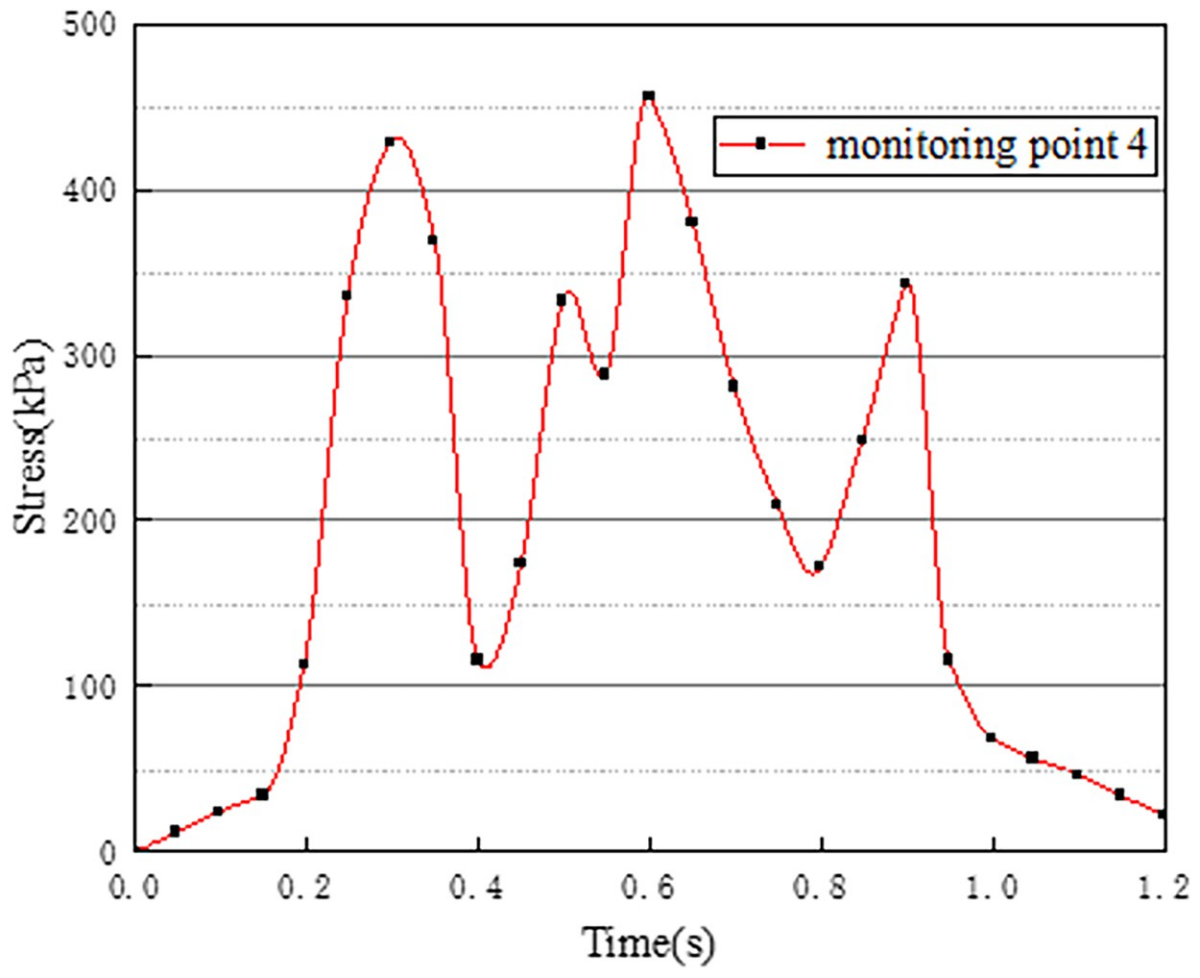
Stress time history curve of monitoring point 4.

In working condition one, the southbound lane is the vehicle congestion situation, the vehicle is traveling slowly, and the northbound lane is the normal vehicle. The effect of vehicle load on the vertical stress of the pre-disintegrated carbonaceous mudstone embankment surface under the condition of unilateral congestion is studied. The stress time history curves of four different monitoring points are shown in Figs 7–10.

It can be seen from Figs 7–10 that in the case of vehicle congestion in the southbound lane, the vertical stress generated by the embankment when the vehicle is running is the same as the vertical stress generated by the embankment under static load. In addition, the maximum increase in vertical stress on the surface of the embankment on the normal driving side is about 170 kPa compared to condition one. The embankment of the northbound lane is not only affected by the vehicles in the other lanes but also affected by the vehicle load in the lane, and stress superimposition has occurred. By comparing the stress time history curves of each measurement point, it can be seen that the impact of the traffic load on the stress of the top surface of the embankment is related to the load and the speed of the vehicle load. The greater the load, the slower the driving speed, and the greater the influence of the driving load on the vertical stress of the embankment.

### 3.3 Analysis of stress change of embankment and monitoring points under the action of vehicle load in working condition 3

**Fig 11:**
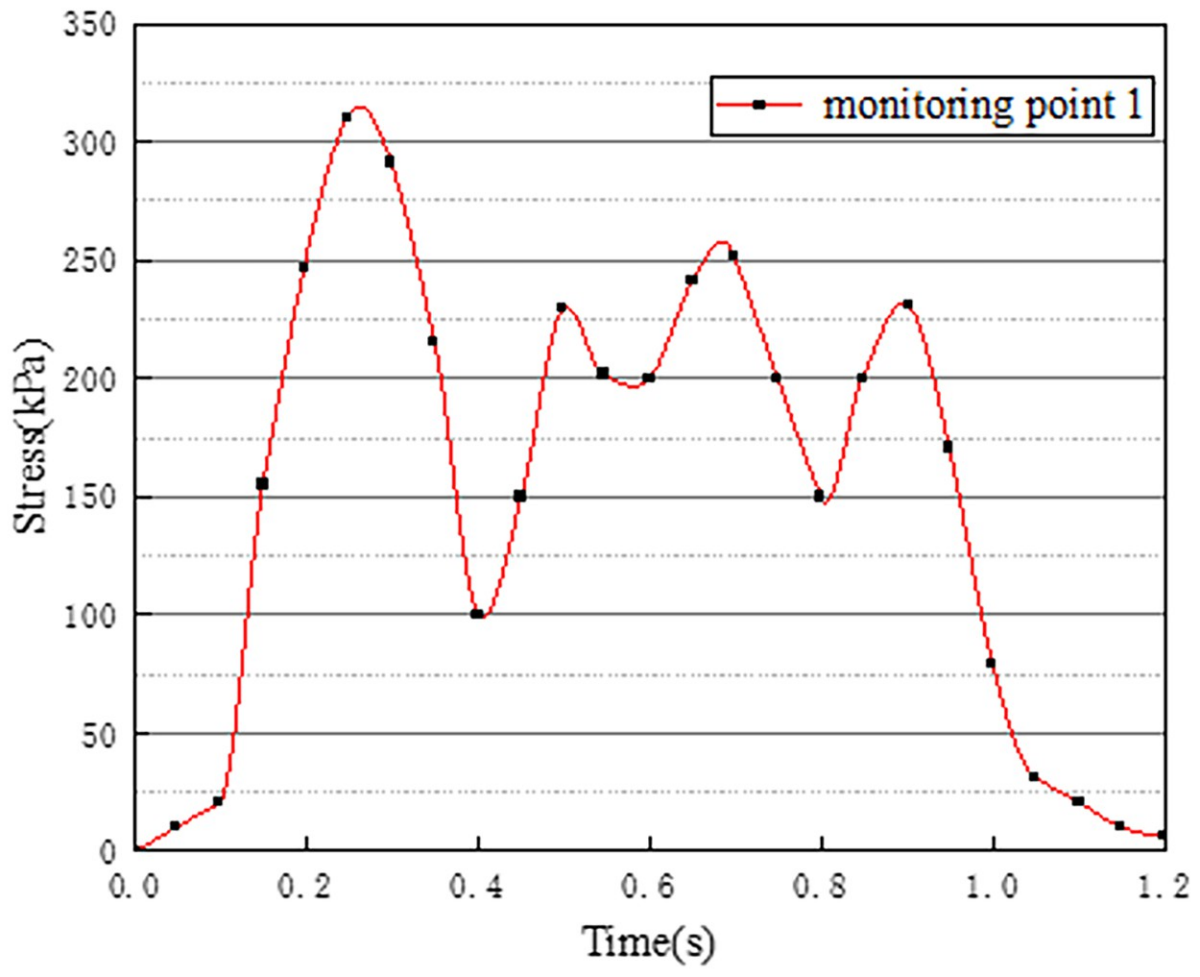
Stress time history curve of monitoring point 1.

**Fig 12:**
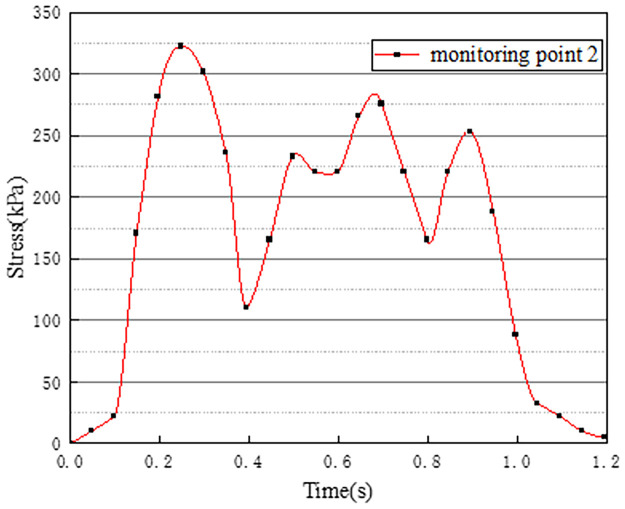
Stress time history curve of monitoring point 2.

**Fig 13:**
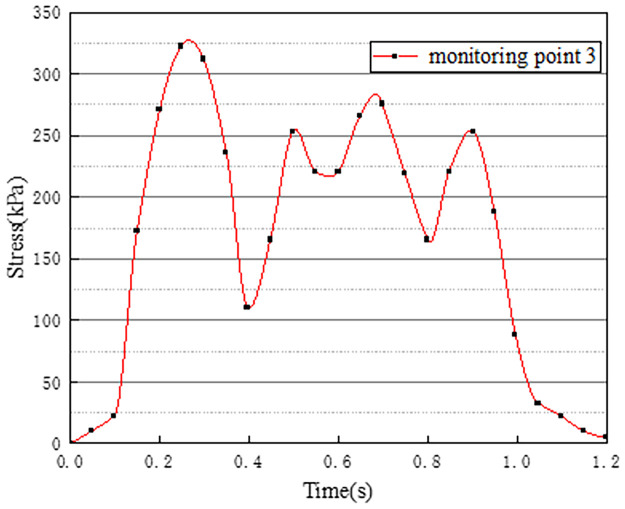
Stress time history curve of monitoring point 3.

**Fig 14:**
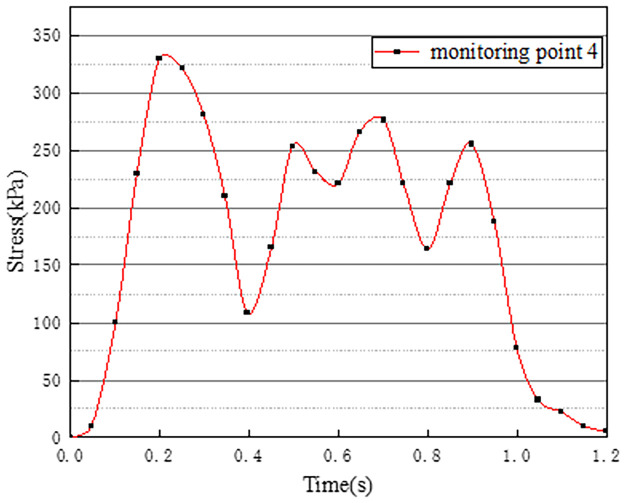
Stress time history curve of monitoring point 4.

Vehicles in two-way lanes in working condition 3 are all driving normally. Under normal driving conditions on both sides, the effect of vehicle load on the vertical stress on the surface of the pre-disintegrated carbonaceous mudstone embankment is studied. The stress time history curves of four different monitoring points are shown in Figs 11–14.

It can be seen from Figs 11–14, in working condition 3, the trend and magnitude of vertical stress time history curves of the four monitoring points are basically the same in the whole vehicle driving process. By comparing with monitoring points 3 and 4 in working condition one, it is found that the vertical stress on monitoring point 3 in working condition three is basically above 150kPa at 0.4s-0.7s, while monitoring point 3 in the working condition one fluctuates. This is because the embankment of the lane is not only affected by the vehicle load of the own lane but also affected by the load of the adjacent lanes. A similar situation occurred at monitoring point 4. The state of the vehicle driving in the northbound lane in working condition three is the same as the setting in working condition one. Although the maximum stress of monitoring points 3 and 4 in working condition 3 is slightly smaller than that of monitoring points 3 and 4 in working condition 1. However, in working condition three, due to the action of normal vehicle loads on both sides, the middle segment of stress time history curves of monitoring points 3 and 4 tends to be more stable and is slightly larger than that of working condition 1. Therefore, when there is a vehicle load on the adjacent lane, the vertical stress on the surface of the pre-disintegrated carbonaceous mudstone roadbed can be increased.

## 4. Analysis of the influence of vehicle load on embankment displacement under three working conditions

### 4.1 Analysis of embankment displacement changes under the action of vehicle load in working condition 1

**Fig 15:**
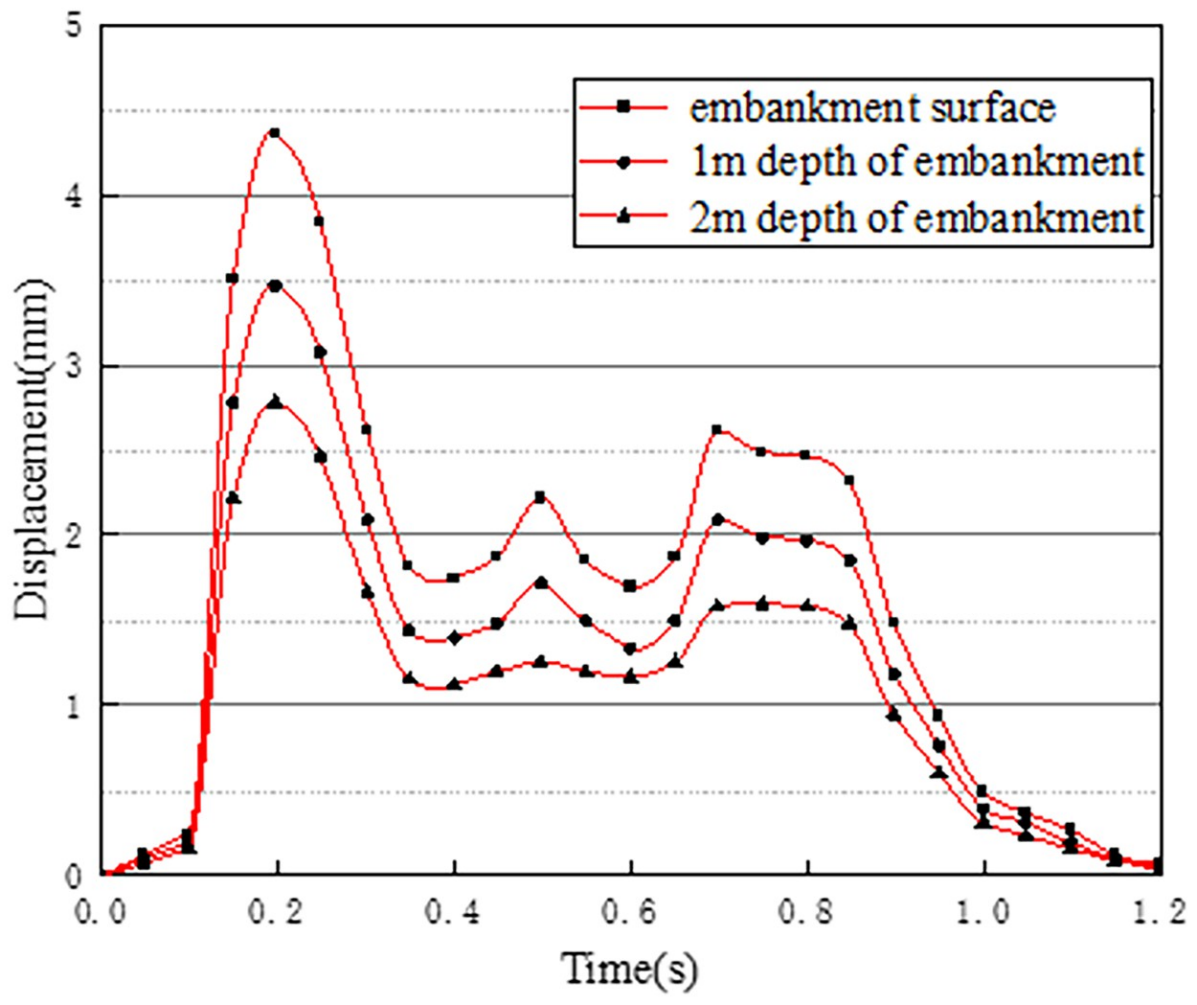
Displacement time history curves at different depths.

The time history curves of vertical displacement at different depths at the center of the pre-disintegrated carbonaceous mudstone embankment are shown in Fig 15.

As can be seen from Fig 15, the maximum vertical displacement at the embankment center can reach 4.33mm when vehicles are driving. According to the displacement time history curves of different depths of the embankment in working condition one, combined with the previous stress analysis at different depths of the embankment, it can be seen that the vertical displacement change of the embankment at the 0–0.6s stage is basically the same as the stress amplitude distribution. The reason is that at this stage, the vehicle is in a state from starting to drive to the embankment section to fully entering the embankment section. At 0.6–0.9s, the displacement time history curve of the embankment surface and the depth of 1m showed obvious hysteresis, and the peak did not appear a cliff-like drop. The reason is that when two vehicles in the front leave the embankment section, the two vehicles in the back come in the same driving state, maintaining the continuity of displacement and settlement. When all vehicles leave the embankment section, the displacement and stress decrease in the same trend at the same time [27]. Analyzing the vertical displacement time history curves at three different depths of the embankment in working condition 1, it can be obtained that the influence of the vehicle load on the vertical displacement of the embankment decreases as the depth of the embankment increases.

### 4.2 Analysis of embankment displacement changes under vehicle load in working condition 2

**Fig 16:**
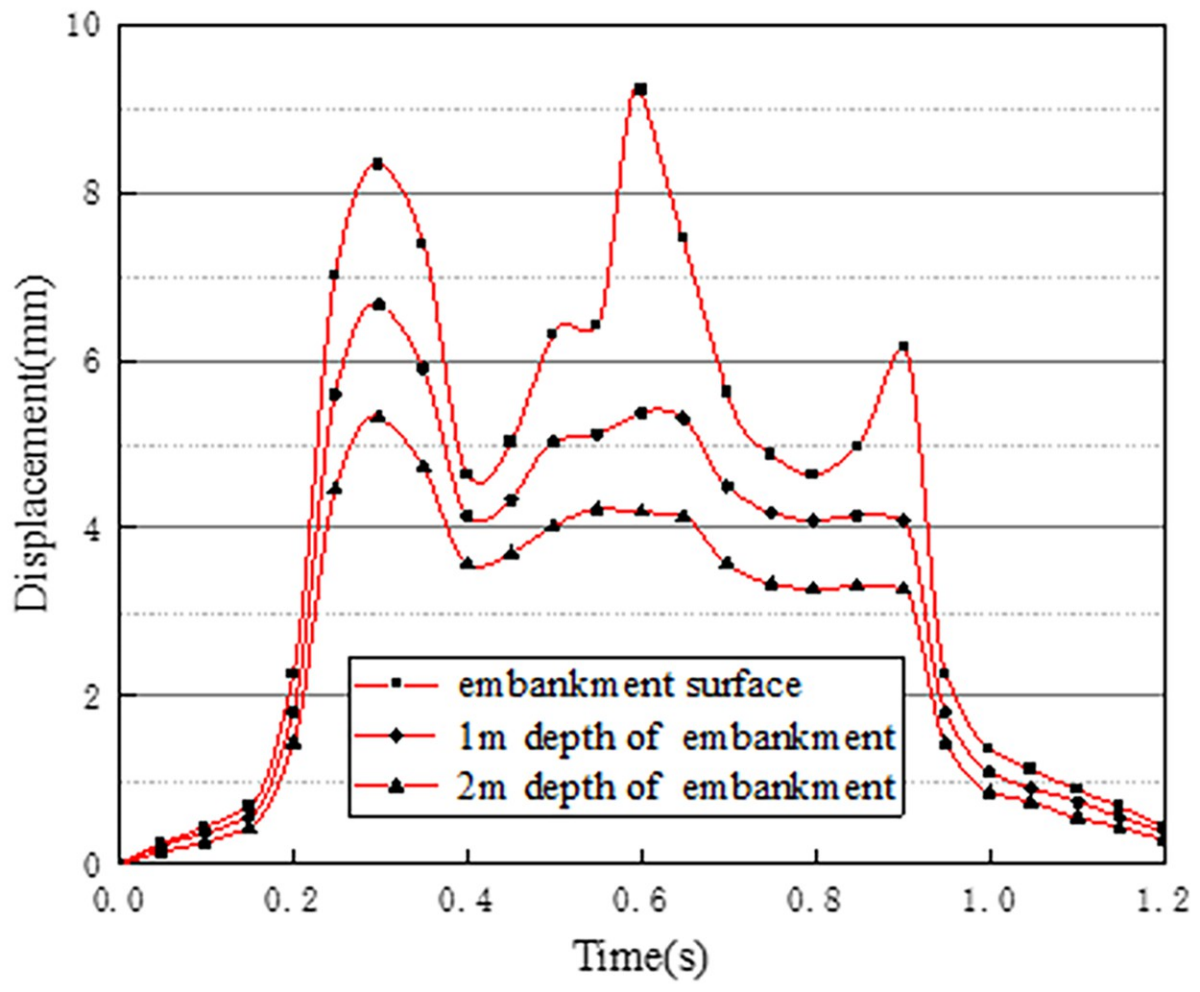
Displacement time history curves at different depths.

The vertical displacement time history curves at different depths at the center of the pre-disintegrated carbonaceous mudstone embankment are shown in Fig 16.

It can be seen from Fig 16 that due to the slow driving of the southbound vehicle, the vehicle load is equivalent to the static load effect, so the vertical displacement value of the pre-disintegrated carbonaceous mudstone embankment is relatively stable during the whole driving process. The displacement at a depth of 2m of the embankment is greater than or equal to the displacement at the surface of the embankment in working condition one and working condition three. Comparing the vertical displacements at different depths of the pre-disintegrated carbonaceous mudstone embankment in working condition one and working condition two, it can be seen that due to traffic jams on the south lane, the vertical displacement at each depth of the embankment has been significantly increased.

### 4.3 Analysis of embankment displacement changes under the action of vehicle load in working condition 3

**Fig 17:**
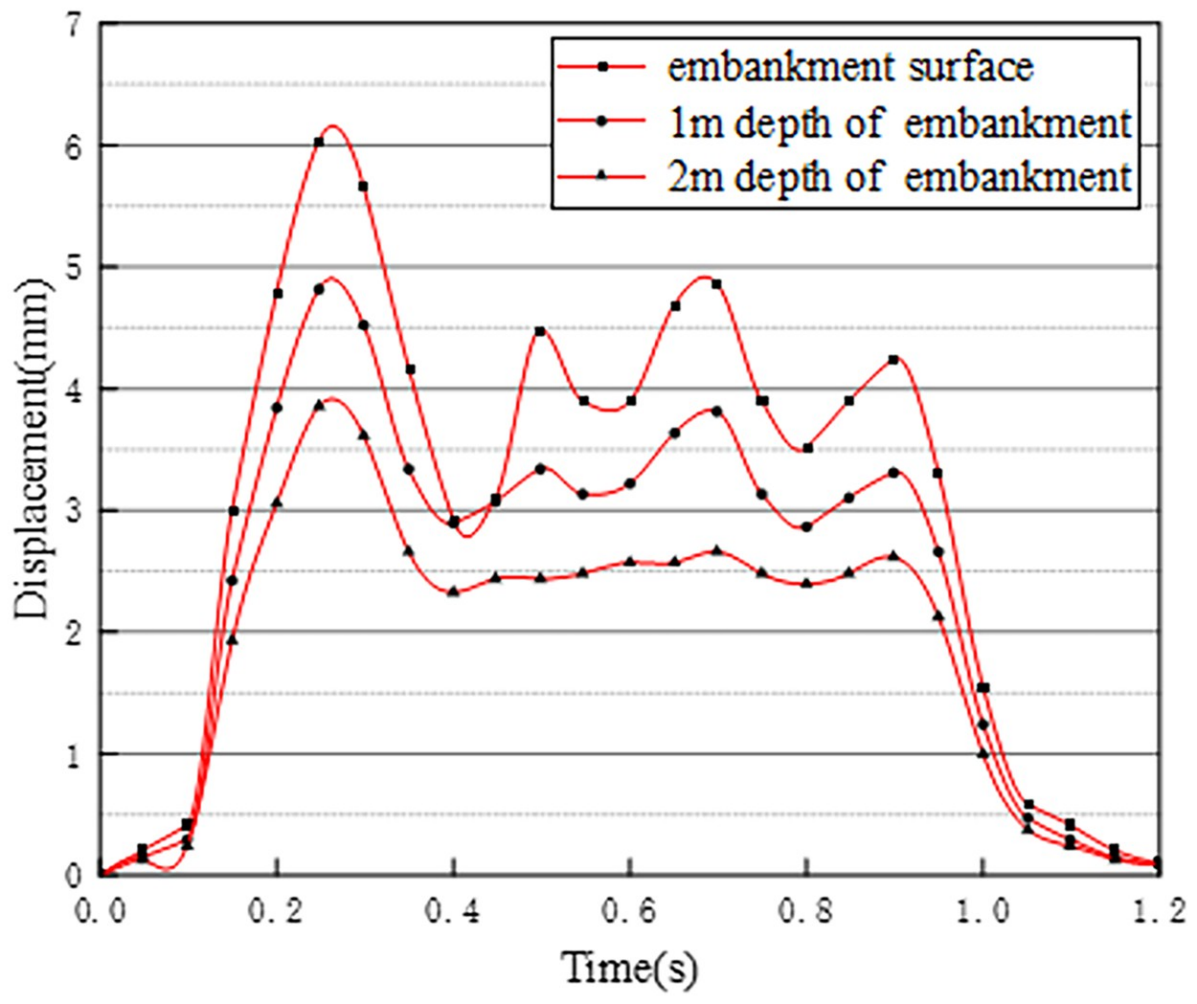
Displacement time history curves at different depths.

The vertical displacement time history curves at different depths at the center of the pre-disintegrated carbonaceous mudstone embankment are shown in Fig 17.

Compared with working condition one, there are vehicles running normally on the two-way lanes of working condition three. It can be found that the displacement at the center of the pre-disintegrated carbonaceous mudstone embankment when the two-way lanes have vehicles in normal driving is larger than the displacement generated when only one-way vehicles are driving. The maximum displacement of the embankment produced by condition three during the entire driving load is 6.02mm, which is an increase of about 1.70mm compared to the maximum vertical displacement of condition one. The reason is that the two-way lanes in working condition three have normal driving vehicle loads, and the traffic load is greater than that of condition one. The changing trend of the displacement time history curve of the embankment surface is basically the same as that of the stress time history curve. However, at a depth of 1m and 2m of the embankment, due to the superimposition of vehicle loads in adjacent lanes and the hysteresis of the displacement of the soil layer, the displacement remains basically unchanged at 0.4s-0.9s.

## 5. Conclusions

1. When only one side has vehicles running normally, the side without vehicle load will also be affected by the vehicle load in the adjacent lane. The closer the monitoring point is to the vehicle load in the adjacent lane, the greater the stress. In addition, the stress change law is basically the same for the lane without vehicles and the lane with vehicles.
2. When the road structure on one side of the vehicle congestion, on the other side of the normal vehicle driving, the maximum increase in vertical stress on the embankment surface in the normal driving side can be about 170 kPa. In addition, the presence of vehicle congestion on one side can increase significantly the vertical displacement at each depth of the embankment. The effect of traffic load on the maximum surface stress of the embankment is related to the vehicle load and the vehicle speed. The larger the load and the slower the speed, the greater the vertical stress on the embankment caused by the vehicle load.
3. The middle part of the stress time curve of monitoring points 3 and 4 in working condition three is more stable and significant than in working condition one. During the whole driving load period, the maximum displacement of working condition three is 6.02mm, which is about 1.70mm higher than the maximum vertical displacement of working condition one. When there is a vehicle load on the adjacent lane, the vertical stress on the surface of the pre-disintegrated carbonaceous mudstone embankment on this side will increase.
4. In this paper, only ABAQUS numerical software is used to study the dynamic response of multi-lane pre-disintegrated carbonaceous mudstone embankments under vehicle load. In the next stage, the dynamic response of multi-lane pre-disintegrated carbonaceous mudstone embankment under vehicle load will be studied by an indoor model test.

## Supporting information

S1 Dataset Experiment data for all cases. (XLSX)
